# Functional Genetic Variations at the microRNA Binding-Site in the *CD44* Gene Are Associated with Risk of Colorectal Cancer in Chinese Populations

**DOI:** 10.1371/journal.pone.0127557

**Published:** 2015-05-26

**Authors:** Xiao-Min Wu, Hong-Guo Yang, Bo-An Zheng, Hong-Feng Cao, Zhi-Ming Hu, Wei-Ding Wu

**Affiliations:** 1 Department of Anesthesiology, Zhejiang Provincial People’s Hospital, Hangzhou, Zhejiang Province, People’s Republic of China; 2 Department of Hepatobiliary and Pancreatic Surgery, Zhejiang Provincial People’s Hospital, Hangzhou, Zhejiang Province, People’s Republic of China; 3 Department of General Surgery, Haining Branch of Zhejiang Provincial People’s Hospital, Jiaxing, Zhejiang Province, People’s Republic of China; 4 Department of Colo-Rectal Surgery, Zhejiang Provincial People’s Hospital, Hangzhou, Zhejiang Province, People’s Republic of China; Medical College of Soochow University, CHINA

## Abstract

CD44 as one of the most putative stem cell markers plays a key role in many cellular processes, including cancer cell growth and migration. Functional single nucleotide polymorphisms (SNPs) of *CD44* may modulate its gene functions and thus cancer risk. In the current study, we investigated if polymorphisms in the 3’-untranslated region (UTR) of *CD44* are associated with increased susceptibility to colorectal cancer (CRC) by conducting a case-control study of 946 CRC patients and 989 cancer-free controls. Three polymorphisms (rs13347C/T, rs10836347C/T, rs11821102G/A) in the 3’-UTR of *CD44* were genotyped. We found that the variant genotypes (CT and TT) of rs13347 (adjusted odds ratio (OR)=1.79, 95% confidence interval (CI)=1.50-2.17) increased an individual’s susceptibility to CRC, compared with rs13347CC homozygous genotypes. We also found that CRC patients with the CT/TT genotype had a 1.6-fold increased risk for developing advanced (stage III + IV) CRC. Furthermore, functional assays showed that the C to T base change at rs13347C/T disrupts the binding site for the microRNA hsa-mir-509-3p, thereby increasing *CD44* transcriptional activity and expression level. These findings suggest that the rs13347C/T in microRNA binding site may be potential biomarkers for genetic susceptibility to CRC.

## Introduction

Colorectal cancer (CRC) is the third most commonly diagnosed gastrointestinal tract worldwide [1], and its incidence and mortality has been rapidly increasing over the past several decades in China [2]. Epidemiological studies have established that environmental risk factors as well as lifestyle-related factors such as dietary, smoking and alcohol drinking are considered as contributors in the etiology of CRC [3, 4]. More and more studies have already recognized that genetic factors may significantly modulate the susceptibility to colorectal cancer. In particular, single nucleotide polymorphisms (SNPs) in genes alter its expression or activity by changing the amino acid sequence may predispose to CRC tumorgenesis [5–7].

Cancer is a class of diseases characterized by uncontrollable cell growth and divide. As a small population of cells within a tumour, cancer stem cells (CSC) could contribute to the most aggressive forms of the disease with capable of initiating tumour growth and their drug resistance properties. Accumulating studies have focused on the existence of colorectal cancer stem cells in human colorectal cancer [8–10]. Thereby, precise identification of colon CSC and their properties can help to significantly advance efficient cancer therapy. Putative colon CSC populations may be identified by the expression of specific CSC markers. CD44 is one of the well-known stem cell marker for CRC [11]. *CD44* gene encoded a cell surface glycoprotein involved in many biological processes including lymphocyte activation, hematopoiesis, homing and embryonal development [12]. Meanwhile, *CD44* plays an indispensable role in tumor cell growth, differentiation, invasion and motility in response to a cellular microenvironment, thereby enhancing cellular aggregation and contributing to the development and progression of tumors [13–15]. Recent studies have shown the significant correlation between level of *CD44* expression and breast cancer cell higher tumorigenicity and metastatic potential [13, 16], thereby highlighting an important role of *CD44* in tumor progression and metastasis. Correspondingly, knockdown of *CD44* with specific siRNA (small interfering RNA) in human colon cancer cells could dramatically suppresse cell growth and tumor progression *in vitro* and *in vivo*, strongly implying a potent regulator of *CD44* in the progression of CRC [17, 18]. In addition, further studies have also stated that genetic variants in the *CD44* gene were associated with cancer risk and prognosis [19, 20]. The 3’—UTRs (untranslated regions) of genes are the main regions targeted by microRNAs and have a central role role in gene’s mRNA stability and eventual modulate the regulation of related proteins. SNPs located at the microRNA-binding sites may affect the binding ability of microRNA and theoretically disturb the expression of *CD44* and thus may predisposite to the disease susceptibility. We hypothesize that SNPs in the *CD44* 3’—UTR are associated with CRC risk by affecting gene’s expression.

In the current hospital—based case—control study, we genotyped three polymorphisms (rs13347C/T, rs10836347C/T, rs11821102G/A) in the 3’–UTR of *CD44* and analyzed the association between the genetic variations and CRC risk. Subsequently functional assays were further performed to investigate the importance and biologic functions of these SNPs.

## Materials and Methods

### Study population

The study population consisted of 946 Han-Chinese CRC patients and 989 ethnically matched cancer-free controls. In the study, colorectal cancer patients or healthy controls who recently had blood transfusions were excluded. The cancer patients with histopathological confirmed CRC consecutively recruited at Zhejiang Provincial People’s Hospital (Hangzhou). Moreover, there were no age, stage and histology restriction for colorectal cancer patients. Meanwhile, all healthy individuals randomly recruited in the study had no documented history of cancer and sex frequency-matched cancer-free controls based on their age (±5 years). They were also randomly recruited from a 3500 individual nutritional survey conducted in Zhejiang Province in the nearly same period, in which the cancer patients’ blood samples were collected, with a response rate of 90%. The tumor, node, metastasis (TNM) classification and tumor staging was evaluated according to the 2002 American Joint Committee on Cancer staging system. Informed consent was signed from each participant for the analysis of molecular correlates, and each participant was scheduled for an interview with selected information such as smoke status, alcohol consumption, family history and other potential confounding factors. The distributions of selected clinical features of the case-control status are summarized in Table 1. 5ml blood samples were collected at enrollment for each of the subjects. This study was approved by the medical ethics committee of Zhejiang Provincial People’s Hospital.

**Table 1:** The distributions of characteristics in control subjects and patients with colorectal cancer from the Chinese population.

Characteristics	Patients (946)		Controls (989)	
	N	(%)	N	(%)
**Median age (range)**	55	(20–90)	58	(21–90)
**Age(years)**				
≤60	509	(53.81)	523	(52.88)
>60	437	(46.19)	466	(47.12)
**Sex**				
Male	519	(54.86)	535	(54.10)
Female	427	(45.14)	454	(45.90)
**Smoking Status**				
Positive	366	(38.69)	399	(40.34)
Negative	580	(61.31)	590	(59.65)
**Alcohol consumption**				
Positive	489	(51.69)	525	(53.08)
Negative	457	(45.88)	464	(46.92)
**Body mass index** (BMI)				
≤20	202	(21.35)	221	(22.35)
20<BMI<28	682	(72.09)	723	(73.10)
≥28	62	(6.55)	45	(4.55)
**Family History of Cancer** *				
Positive	96	(10.15)	116	(11.73)
Negative	850	(89.85)	873	(88.27)
**TNM stage**				
I	84	(8.88)		
II	262	(27.70)		
III	344	(36.36)		
IV	256	(27.06)		

*Family history of all malignant tumors in first degree relatives.

### Genotyping analysis

Genomic DNA was extracted from each blood sample obtained from all participants by using a Blood Mini Kit (Qiagen, Valencia, CA) and stored at -80°C. Three polymorphisms (rs13347C/T, rs10836347C/T, rs11821102G/A) in the 3’-UTR of *CD44* were selected and genotyped by Allele-specific MALDI-TOF mass spectrometry analysis as previously described [21]. Primers pairs and multiplex reactions were designed by RealS NP.com Website. Approximately 10% MALDI-TOF mass spectrometry analyzed samples were randomly selected for a blinded repeat. For quality control purposes, 50 samples were reanalyzed by direct sequencing and the results were in 100% agreement.

### Construction of luciferase reporters

**Fig 1:**
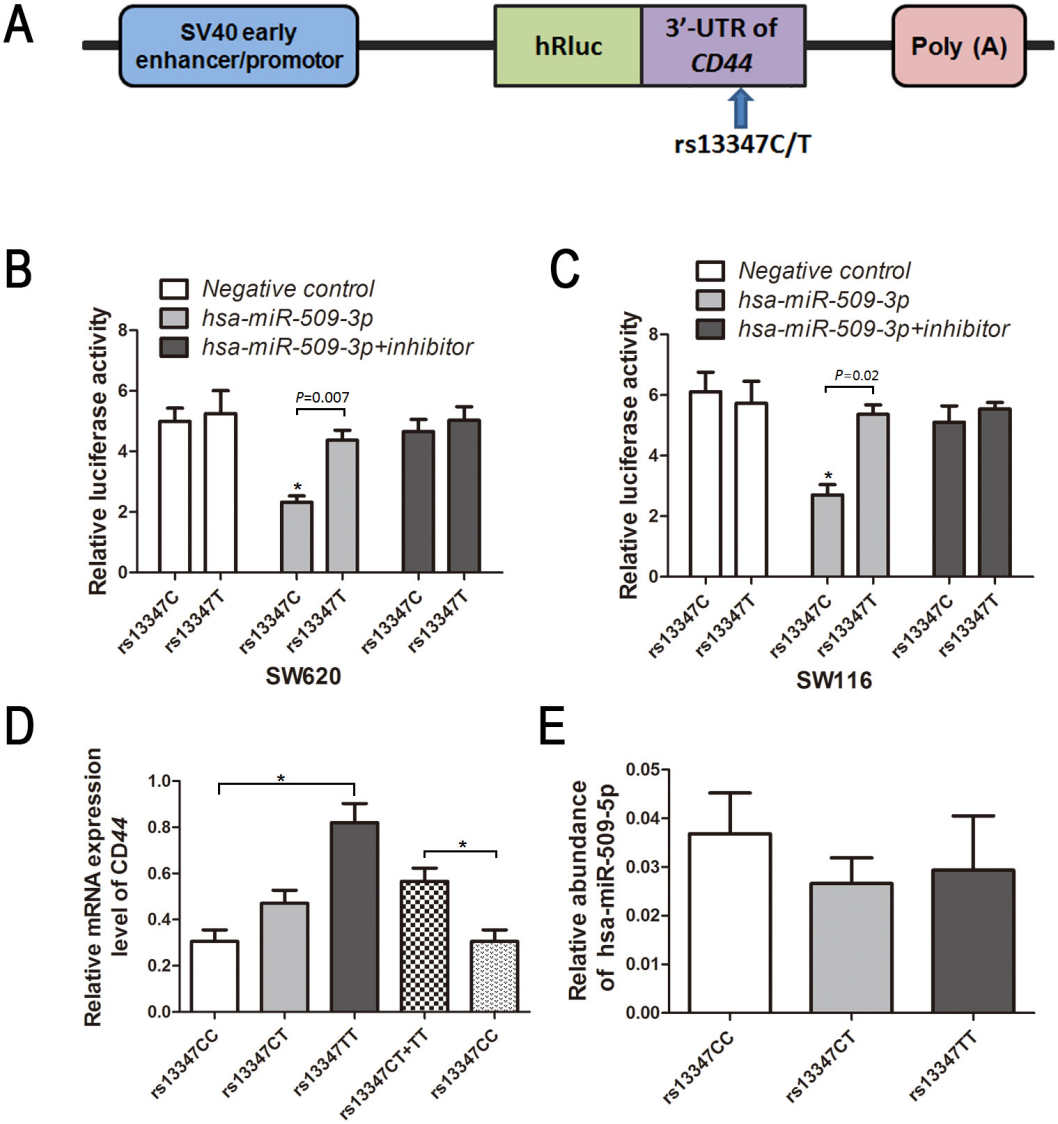
**(A)** Representative graph of luciferase activity of variant allele on luciferase reporter genes bearing the 358bp of 3’-UTR fragment of the human *CD44* gene flanking the rs13347C/T polymorphism. Relative luciferase activity of the psiCHECK2-*CD44*-rs13347C and psiCHECK2-*CD44*-rs13347T constructs cotransfected with or without 40pmol hsa-mir-509-3p or 40pmol hsa-mir-509-3p inhibitor, respectively performed in SW260 cells **(B)** and SW116 cells **(C)**. Six replicates were carried out for each group, and the experiment was repeated at least three times. Asterisk indicates a significant change (*P*<0.05). Data are mean±SEM. **(D)** *CD44* expression level in 37 CRC tissues from individuals with different rs13347C/T genotypes (15 rs13347CC, 16 rs13347CT and 6 rs13347TT); *CD44* expression was normalized to the *GAPDH* gene and presented as mean ± SEM. Data shown are representative of at least three independent experiments. **(E)** The expression level of hsa-miR-509-3p in 37 CRC tissues grouped by rs13347C/T genotypes. The hsa-miR-509-3p mRNA expression was calculated relative to expression of *U6* mRNA by calculating the relative expression levels. Data are mean±SEM, *P* = 0.579. The differences in the expression levels were analyzed with one-way ANOVA test.

Genotyping for *CD44* SNPs showed that rs13347C/T was significantly associated with CRC risk. Based on the bioinformatics analysis (SNPinfo Web server: http://snpinfo.niehs.nih.gov/), the rs13347C to rs13347T transition gained a new binding of the microRNA hsa-mir-509-3p. Therefore, we hypothesised that SNP rs13347C/T at the microRNA-binding sites may influence the binding ability of hsa-mir-509-3p, and thus had any effect on *CD44* expression. To test this hypothesis, the 358bp 3’-UTR fragment of the human *CD44* gene flanking the rs13347C or rs13347T allele was synthesized by the Genewiz Company (Suzhou, China), and were then cloned into the psiCHECK2 basic vector (Promega, Madison, WI, USA) with renilla and firefly luciferase gene sequences (Fig 1A). The two constructs (psiCHECK2-*CD44*-rs13347C and psiCHECK2-*CD44*-rs13347T) were sequenced to confirm the allele and integrity of each insert.

### Transient transfections and luciferase assays

Two human colorectal cell lines SW116 and SW620 were maintained in L-15 medium (Gibco, Los Angeles, California, USA) with 10% fetal bovine serum (Sigma-Aldrich, MO) at 37°C in a humidified atmosphere with 5% CO_2_. 1×10^5^ cells per well were seeded in 24-well plates (BD Bio sciences, Bedford, MA, USA) before transfection. After 24h, the cells were then transiently transfected as described previously [22, 23] with 1.5μg reporter plasmids (rs13347C or rs13347T allele) with or without 40pmol has-mir-509-3p mimics or 40pmol inhibitors by Lipofectamine 2000 according to the protocol (Invitrogen, Carlsbad, CA). For luciferase assays, cells were harvested after transfection for 24h, and renilla luciferase activity was quantified with the Dual-Luciferase Reporter Assay System (Promega, Madison, WI).

### Real-time PCR

RNAs from 37 CRC tissues with different rs13347C/T genotypes were extracted and reverse-transcribed to cDNA using oligo-dT primer. Allelic discrimination of rs13347 polymorphisms of the *CD44* gene was performed with the ABI Prism 7500 sequence detection system to evaluate *CD44* and *GAPDH* mRNA levels based on the SYBR-Green method. Al l quantifications were carried out with *GAPDH* as the internal standard. Additionally, the TaqMan MicroRNA Assays (Applied Biosystems) was used to detect the background expression of has-mir-509-3p in CRC tissues according to the manufacturers’ protocol. Expression of the universally expressed *U6* small nuclear RNA was also used for an internal control. The relative quantification of *CD44* expression was analysed by the 2^-ΔΔCT^ method. Each assay was done in triplicate.

### Statistical analysis

Two-sided chi-square tests were used to compare differences in the distributions of clinicopathological features between the cases and cancer-free controls. For the case-control study, the Hardy—Weinberg equilibrium (HWE) was tested to compare the expected genotype frequencies with observed genotype frequencies in controls by a goodness-of-fit chi-square test. The correlation between genetic variants and clinical parameters was examined using the Chi square test as appropriate. The odds ratios (ORs) and 95% confidence intervals (CIs) were estimated to assess the correlation between rs13347C/T polymorphism and CRC risk using an unconditional logistic regression models. Stratified analysis was also performed to assess the possible interaction between *CD44* polymorphisms and selected variables on CRC risk. Statistical comparisons of more than two groups were evaluated by one-way ANOVA tests. The statistical power was calculated by using the PS Software (http://biostat.mc.vanderbilt.edu/twiki/bin/view/Main/PowerSample-Size). The statistical analyses were conducted using STATA software (version 10; STATA Corporation). The association was considered significant when *P*-value was less than 0.05.

## Results

### Genotypes and risk of CRC

A total of 946 CRC cases and 989 healthy controls were recruited in our analysis and genotyped to assess the associations between *CD44* rs13347C/T, rs10836347C/T, rs11821102G/A and CRC risk. The genotype distributions of the three polymorphisms between the cases and controls are summarized in Table 2. The observed genotype frequencies of these SNPs studied in healthy controls were found to be consistent with HWE. Genotyping results showed that only rs13347 was statistically significantly associated with CRC risk, no significant associations were noted in other SNPs. Patients with the *CD44* polymorphism rs13347TT genotypes was associated with the increased risk of colorectal cancer between the cases and controls. Furthermore, carrier with the combined variant genotype CT/TT exhibited a significantly higher risk of CRC (OR = 1.79, 95% (CI) = 1.50–2.17), *P*<10^−4^), compared with the genotype CC.

**Table 2:** Genotypes frequency of SNPs in 3’-UTR of the *CD44* gene in healthy controls and CRC patients and their association with CRC risk.

Genotypes	Cases (946)		Controls (989)		Adjusted OR (95% CI)^a^	*P*_value_ ^b^
	N	(%)	N	(%)		
**rs13347C/T**						
CC	416	(43.97)	578	(58.44)	1.00 (Reference)	
CT	441	(46.62)	348	(35.19)	1.78 (1.46–2.16)	**<10** ^**−4**^
TT	89	(9.41)	63	(6.37)	1.97 (1.38–2.83)	
CT+TT	530	(56.03)	411	(41.56)	1.79 (1.50–2.17)	
Allele frequency						
C	1273	(67.28)	1504	(76.04)	1.00 (Reference)	
T	619	(32.72)	474	(26.36)	1.54 (1.37–1.79)	**<10** ^**−4**^
**rs10836347C/T**						
CC	821	(86.79)	851	(86.05)	1.00 (Reference)	
CT	120	(12.68)	129	(13.04)	0.88 (0.67–1.15)	0.506
TT	5	(0.53)	9	(0.91)	0.57 (0.19–1.89)	
CT+TT	125	(13.21)	138	(13.95)	0.95 (0.73–1.26)	
Allele frequency						
C	1762	(93.13)	1831	(92.57)	1.00 (Reference)	
T	130	(6.87)	147	(7.43)	0.92 (0.71–1.20)	0.499
**rs11821102G/A**						
GG	815	(86.15)	843	(85.24)	1.00 (Reference)	
AG	119	(12.58)	131	(13.25)	0.94 (0.73–1.26)	0.523
AA	12	(1.27)	15	(1.52)	0.83 (0.37–1.89)	
AG+AA	131	(13.85)	146	(14.76)	0.92 (0.71–1.22)	
Allele frequency						
G	1749	(92.44)	1817	(91.86)	1.00 (Reference)	
A	143	(7.56)	161	(8.14)	0.92 (0.72–1.16)	0.502

^a^Data were calculated by unconditional logistic regression, adjusted for age, sex, BMI and family history of CRC.

^b^Tests for trend of odds were two-sided.

We further performed the stratified analysis for rs13347C/T by clinical or pathologic characteristics such as age, sex, smoking status, alcohol consumption and family history of cancer. As shown in the Table 3, there was a significant interaction between the polymorphism rs13347C/T and tumor stage. We observed that compared with the rs13347CC genotype, patients with the CT/TT genotype had over 1.6-fold increased risk for developing advanced (stage III+IV) CRC (*P* = 0.004). However, no differences in other subgroups were found.

**Table 3:** Stratified analysis for associations between the *CD44* rs13347C/T polymorphism and CRC risk.

Variables	Cases (946)				Controls (989)				Adjusted OR (95%CI)^a^	*P*_value_ ^b^
	CC(N)	(%)	CT+TT(N)	(%)	CC(N)	(%)	CT+TT(N)	(%)	CC *VS*.CT+TT	
**Age (years)**										
≤60	229	(24.21)	280	(29.60)	308	(31.14)	215	(21.74)	1.75 (1.37–2.24)	
>60	187	(19.77)	250	(26.43)	270	(27.30)	196	(19.82)	1.84 (1.41–2.40)	0.78
**Sex**										
Male	291	(30.76)	228	(24.10)	386	(39.03)	149	(15.07)	2.03 (1.57–2.61)	
Female	125	(13.21)	302	(31.92)	192	(19.41)	262	(26.49)	1.77 (1.34–2.34)	0.48
**Smoking Status**										
Positive	235	(24.84)	131	(13.85)	322	(32.56)	77	(7.79)	2.33 (1.67–3.25)	
Negative	181	(19.13)	399	(42.18)	256	(25.88)	334	(33.77)	1.69 (1.33–2.15)	0.12
**Drinking Status**										
Positive	209	(22.09)	280	(29.60)	284	(28.72)	241	(24.37)	1.58 (1.25–2.02)	
Negative	207	(21.88)	250	(26.43)	294	(29.73)	170	(17.19)	2.09 (1.62–2.78)	0.13
**Body Mass Index**										
≤20	94	(9.94)	108	(11.42)	145	(14.66)	76	(7.68)	2.11 (1.46–2.47)	
20<BMI<28	271	(28.65)	411	(43.45)	397	(40.14)	326	(32.96)	1.85 (1.51–2.27)	0.22
≥28	51	(5.39)	11	(1.16)	36	(3.64)	9	(0.91)	0.89 (0.33–2.32)	
**Family History of Cancer** *										
Positive	64	(6.77)	32	(3.38)	89	(9.00)	27	(2.73)	1.65 (1.11–3.03)	
Negative	352	(37.21)	498	(52.64)	489	(49.44)	384	(38.83)	1.82 (1.51–2.21)	0.78
**TNM stage**										
I/II	178	(18.82)	168	(17.76)	578	(58.44)	411	(41.56)	1.33 (1.04–1.70)	
III/IV	238	(25.16)	362	(38.27)	578	(58.44)	411	(41.56)	2.14 (1.74–2.64)	**0.004**

^a^Adjusted for age, sex, smoking status, drinking status and family history of cancer in a logistic regression model where it was appropriate.

^b^Two-sided x^2^ test for the ORs obtained from the multivariate logistic regression. A *P*_value_ of less than 0.05 was considered to be statistically significant.

*Family history of all malignant tumors in first degree relatives.

### Effect of the SNP rs13347C/T on reporter gene’s activity

Two luciferase reporter constructs containing rs13347C or rs13347T allele transiently were co-transfected with mimic or inhibitor of hsa-mir-509-3p that were predicated binding to rs13347C/T polymorphic site by bioinformatics analysis to investigate the effect of SNP rs13347C/T on *CD44* 3’-UTR transcription activity. As shown in Fig 1B and 1C, the microRNA hsa-mir-509-3p mimic transfected CRC cells could significantly decrease the luciferase activity of reporter gene with the rs2735383C allele compared with rs2735383T allele (*P* = 0.007 for SW620 and *P* = 0.02 for SW116). In contrast, the activity of reporter gene with the rs13347C allele significantly trended to reverse following transient transfection of cells with hsa-mir-509-3p mimic and its corresponding inhibitor. However, no any significant effects on the reporter genes with the rs2735383T allele with treatment of the hsa-mir-509-3p mimic and inhibitor was observed. These results indicated that the C to T base change of rs13347C/T disrupts the binding site for hsa-mir-509-3p, thereby increased the transcriptional activity of the *CD44* gene.

### Association between rs13347C/T genotypes and *CD44* expression

We carried out real-time PCR to evaluate the effects of rs13347C/T on *CD44* expression using 37 tumor tissues from untreated CRC patients with different genotypes. As illustrated shown in Fig 1D, the results revealed that patients with rs13347T genotypes (CT and TT) harbored a significantly higher *CD44* mRNA levels (mean±standard error of the mean (SEM): 0.565±0.057), compared to carriers of the rs13347CC genotypes (mean±SEM:0.305±0.050; ANOVA test: *P* = 0.003). And we then analyzed the association between hsa-mir-509-3p expression and *CD44* rs13347C/T genotypes. The hsa-mir-509-3p is constitutively expressed in the 37 tumor tissues from untreated CRC patients with different genotypes; however, there was no significant association between the background expression of hsa-mir-509-3p and the *CD44* rs13347C/T genotypes (0.037±0.008 for CC; 0.027±0.005 for CT and 0.029±0.011 for TT; ANOVA test: *P* = 0.579) (Fig 1E).

## Discussion

In this present molecular epidemiological study, we investigated the associations of three SNPs in the 3’-UTR of *CD44* gene with risk of colorectal cancer in Chinese population and found that the rs13347C/T was significantly associated with an increased risk of CRC. We also observed a significant elevated risk of CRC tumor stage associated with the rs13347T variant in an allele-dose manner. Further functional experiments showed that the rs13347T variant can significantly alter hsa-mir-509-3p-mediated *CD44* transcriptional activity and expression activity. The results indicate that variants in *CD44* gene may be valuable for risk assessment, diagnosis and genetic epidemiological analysis of CRC.

The *CD44* gene is located to chromosomal locus 11p13 and encodes a widely expressed cell surface transmembrane glycoprotein in a variety of cell types. The protein mainly encompasses with N terminus (residues 38–46) and C-terminal portion of the extracellular domain (residues 150–162), which is known to be necessary for binding a diverse repertoire of ligands such as hyaluronic acid (HA), osteopontin and matrix metalloproteinase [24]. There are several lines of experimental evidence supporting a role for *CD44* involved in a vast range of cellular processes including lymphocyte activation, apoptosis, hematopoiesis, recirculation and homing [25–27]. *CD44* has a well-documented tumor-promoting activity that includes promoting tumor cell growth, differentiation and metastasis [28]. Many molecular biology studies have identified that *CD44* expression is closely related to the various malignancies occurrence, progression and prognosis [29–32]. Unexceptionally, *CD44* was also found to play an important role in CRC development. A recent study based on 234 CRC patients have demonstrated that the increased *CD44* expression in CRC is correlated with metastasis, increased early tumor recurrence and chemoresistance [33]. Recent experimental evidence from both *in vitro* clonogenic and *in vivo* tumorigenic assay revealed that CD44 (+) CRC cells display the properties of cancer stem cell [18], indicating a functional importance of *CD44* in the CRC initiation and development. Consistently, we have established genetic association between the expression of *CD44* and risk of CRC. Additionally, functional analyses showed that the *CD44* rs13347C/T SNP may affect *CD44* expression by modifying hsa-mir-509-3p binding to the 3’—UTR of the *CD44* gene. Previous studies have reported that hsa-mir-509-3p may play an important role as a tumor suppressor gene during cancer formation [34, 35]. And no significant association between the background expression of hsa-mir-509-3p and the *CD44* rs13347C/T genotypes was observed in the study, indicating that some unrecognized mechanisms modulating the effect of hsa-mir-509-3p on rs13347T genotypes may exist. Our result showed that the C to T base change of rs13347C/T possibly disrupts the binding site for hsa-mir-509-3p, increases the transcriptional activity of the *CD44* gene and thus was more susceptible to CRC.

In the present study, we first demonstrated that *CD44* rs13347C/T variation contributes to an increased risk of CRC. The current study is based on a relatively large, random, ethnically homogenous sample, meaning that it is possible to avoid any confounding bias. Of note, the statistical power was increased to be 0.91 (two-sided test, α = 0.05) in detecting an OR of N for the rs13347CT+TT genotypes (occurring at a frequency of 41.56% amongst the controls) compared with the rs13347CC genotype. In addition, the association is biologically plausible, suggesting this finding is noteworthy.

In conclusion, this is the first report to find association between polymorphism rs13347C/T located in the hsa-mir-509-3p binding site, which may affect *CD44* mRNA expression level and the risk of CRC. Additionally, our study indicated that compared with the CD44 rs13347CC genotype, the variant genotypes (CT+TT) confers an increased risk of CRC populations. Moreover, the risk effect of this polymorphism is more obvious in tumor stage (III+IV) of CRC patients. Larger, preferably population-based case-control studies are necessary to confirm the associations between *CD44* polymorphisms with different human malignancies in different ethnic groups.
